# Complexities of Prostate Cancer

**DOI:** 10.3390/ijms232214257

**Published:** 2022-11-17

**Authors:** Sobia Wasim, Sang-Yoon Lee, Jaehong Kim

**Affiliations:** 1 Department of Neuroscience, College of Medicine, Gachon University, Incheon 21936, Republic of Korea; 2 Department of Biochemistry, College of Medicine, Gachon University, Incheon 21999, Republic of Korea

**Keywords:** prostate cancer, PSA, resistance, mutation, AR signaling

## Abstract

Prostate cancer has a long disease history and a wide variety and uncertainty in individual patients’ clinical progress. In recent years, we have seen a revolutionary advance in both prostate cancer patient care and in the research field. The power of deep sequencing has provided cistromic and transcriptomic knowledge of prostate cancer that has not discovered before. Our understanding of prostate cancer biology, from bedside and molecular imaging techniques, has also been greatly advanced. It is important that our current theragnostic schemes, including our diagnostic modalities, therapeutic responses, and the drugs available to target non-AR signaling should be improved. This review article discusses the current progress in the understanding of prostate cancer biology and the recent advances in diagnostic and therapeutic strategies.

## 1. Introduction

The prostate, which is an accessory reproductive organ in men, is located below the bladder. Its major function is to complement the essential secretions to semen and to keep the sperm viable. The adult human prostate is divided into central, transitional, and peripheral regions. More than 95% of prostate cancer (PCa) cases are adenocarcinomas, most of which have an acinar origin, while few have a ductal origin. Almost 80% of prostate adenocarcinomas arise from the luminal or the basal (with a lesser prevalence) epithelial cells in the peripheral regions, which occupy >70% of the total prostate tissue. The prevalence of PCa in men who are aged >65 years is approximately six out of ten cases. It is more frequent among Caribbean men of African ancestry and among African American men than among men of other races; however, the reason for this remains unclear. Due to its high prevalence, PCa is the second most diagnosed solid-organ cancer, after lung cancer, in men [1,2] and it is also a major health issue, with 358,989 identified deaths globally and approximately 1.3 million newly diagnosed cases in 2018 [3]. Worldwide, approximately 10 million men are currently living with the disease, and approximately 700,000 of them have a metastatic form of the disease [4]. Although PCa is generally diagnosed at an early stage, the risk–benefit ratio of the treatment remains uncertain. It is one of the most disputed areas of medicine because of the significant morbidity from the current form of therapy [5,6]. Because of its long disease history and uncertainty in individual patients’ clinical progress, clinicians tend to consider the treatment workup of PCa as a long journey [7]. 

## 2. Prognosis of Prostate Cancer

PCa is a highly heterogeneous complex cancer that shows widely varying levels of mortality and morbidity. Among PCa cases, adenocarcinomas that have an acinar origin have a far better prognosis than those with a ductal origin. Approximately 80% of men who are diagnosed with PCa are diagnosed with prostate-limited localized PCa [8]. 

If it is diagnosed at an early stage, the life expectancy for men with localized PCa can be as high as 99% for more than 10 years [9]. For most men with PCa, managing a customized treatment plan for a slow-growing, and often even indolent, cancer is necessary in order to live with the disease; however, for several others, relapsed PCa following a definitive treatment plan may be aggressive and, in unusual cases, may be unresponsive to the current form of standard care. Approximately 5% of men who are diagnosed with PCa are diagnosed with distant metastases (often in multiple sites), and 15% of them are diagnosed with locoregional metastases [8]. If they are diagnosed with late-stage PCa (distant metastases), men have a poor overall survival rate of only 30% for five years [8]. Metastatic PCa accounts for more than 400,000 deaths annually, and it is expected that this mortality rate will increase by two-fold or more by 2040 [4]. Moreover, it is estimated that, after diagnosis, a similar number of men will live with treatment-related morbidity for more than 10 years [4]. The metastasized PCa cells can stay dormant in the tumor microenvironment at a secondary site for a long time. The metastasis of PCa is primarily associated with the spread to the locoregional lymph nodes and/or the hematogenous spread to the stroma of the bone marrow in the axial skeleton [10]. More than 80% of distant metastatic lesions are found in the bone tissue [10]. In more unusual cases, the metastasis of PCa is associated with the spread to distant visceral sites. Almost all patients with metastatic PCa ultimately experience castration-resistant PCa (CRPC), which is refractory to androgen deprivation therapy (ADT). These features are the principal causes of PCa morbidity and mortality [10]. Metastatic CRPC (mCRPC) eventually becomes therapy- and castration-resistant PCa (t-CRPC), which has no further effective solution and is considered to be an end-stage disease [11,12].

## 3. Tumor Heterogeneity

Localized PCa is often found to be morphologically heterogeneous within the same patient. Multiple tumor foci can appear within the prostate organ (intertumoral heterogeneity), and they can have genetic differences that cause various degrees of metastatic spread and treatment resistance [13]. The genomic heterogeneity that can be observed in localized PCa confronts the concept of a “dominant cancer lesion”, which can be largely responsible for a patient’s clinical course. Furthermore, the cancer cells within one focus may arise from different ancestor cells that become individually transformed [14] or from one single clone that transforms and diverges into multiple distinct clones in one focus (intratumoral heterogeneity) [15]. The metastatic PCa that often occurs in multiple locations and is supposed to be clonally derived can harbor multiple subclones that are genetically distinct with different molecular features [16].

The heterogeneity of potential cancer driver genes further complicates the understanding of the clinical profile of PCa at the time when it is diagnosed and the treatment options with the available targeted agents in the future. In the prostate epithelial cells, differentiation and proliferation are dependent on the androgen receptor (AR) activity, and current ADT takes advantage of the PCa’s dependence on the AR activity. ADT and second-line therapies are also believed to increase the heterogeneity [17]. The role of tumor heterogeneity is suspected to be in the progression of PCa during or after standard ADT. Molecular heterogeneity indicates that the genomic features may determine the disease severity and the unresponsiveness to conventional therapy [18]. Current diagnostic prostate biopsy is significantly hampered by this polyclonality, because one large biopsied lesion does not always provide sufficient insights into the other lesions. Mutations comprising the genetic heterogeneity are discussed in Section 5, “Genomics”.

## 4. Our Diagnostic Tools

### 4.1. PSA Testing

Patients with the early stages of PCa do not experience symptoms. The diagnosis and the treatment plans of PCa changed significantly in 1979 when the prostate-specific antigen (PSA) was first described [19]. PSA is a serine protease that is transcriptionally dependent on the AR that is expressed in the epithelial cells of benign hypertrophic and malignant prostatic lesions, but not in other tissues in humans. Although the PCa-related mortality and the number of cases with advanced-stage disease at diagnosis have sharply declined after the application of PSA testing, the common practice of PSA screening results in concerns of the overdiagnosis and the overtreatment of slow-growing or indolent PCa, which can be treated effectively [20]. This is significant considering that, according to a report, >40% of men who were diagnosed with PCa had low-grade cancer that may have never become clinically apparent [21]. Every year, one million men in both the USA and in Europe, respectively, receive secondary care with elevated serum PSA levels (generally ≥3–4 ng/mL) [22,23]. Although the European Randomized Study of Screening for Prostate Cancer (ERSPC), comprising 182,160 men from eight European countries, reported a 27% reduction in PCa-specific mortality, it turned out that 781 men should be screened with PSA testing in order to prevent one PCa-related death [24].

In fact, because the PSA levels can be found to be increased in numerous benign lesions, and its base level is generally subject to differences in the race and the age of men, PSA testing suffers from high false positive findings. This can result in unnecessary invasive diagnostic procedures, which are painful and expensive, being performed on men with mere benign lesions in order to rule out malignancies. It may also result in radical prostatectomies, which are also unnecessary, being performed on a large number of men with low-risk localized and indolent PCa [25]. Because the serum PSA levels are, most often, not specific to clinically significant PCa (CSC), the overdiagnosis of low-risk PCa becomes an important issue [26]. Serum PSA levels can also be elevated in benign prostate hypertrophy and prostate infection; hence, the determination of an elevated serum PSA level (from 3 to 10 ng/mL) must be considered relative to each patient’s baseline level. It is recommended that individuals must check their baseline PSA level at the age of 40 years in order to aid accurate individual future PCa screening [27]. Consequently, informed decision making is recommended for individual PCa testing or screening.

Currently, we do not have an error-free diagnostic modality for distinguishing aggressive PCa from slow-growing or indolent PCa. Because of the lack of reliable imaging tools other than magnetic resonance imaging (MRI), the current standard diagnostic approaches for PCa are the detection of abnormal PSA findings, potentially resulting in digital rectal examination (DRE) to palpate the prostate in order to measure its texture, its stiffness, its enlargement, and the nontargeted transrectal ultrasound (TRUS)-guided biopsy sampling of 10–12 biopsy cores for histopathological diagnosis, which heavily suffers from under sampling and complications.

These approaches are in sharp contrast to those that are applied for most other solid tumors, where the reported symptom results in the identification of lesions, which occurs mostly via imaging, and targeted and guided biopsies are performed on the suspicious lesions. The current diagnostic workup of PCa should aim towards the following points: a reduction in the practice of unnecessary biopsies, an improved detection of CSC, and the avoidance of the overdiagnosis and overtreatment of clinically insignificant PCa.

### 4.2. Gleason Grading System

The sum of the top prominent and the second prominent Gleason pattern number (in which each number is between one and five) is the Gleason score. Historically, the aggressiveness of the PCa has been graded using the Gleason system, in which the microscopic assessment of the histopathological features is used in order to classify the cancer tissue as poorly-differentiated (the highest grade) to well-differentiated (the lowest grade). In 2014, the grading system was reorganized into the International Society of Urological Pathology (ISUP) grade groups 1–5 [28]. The risk assessment of PCa at diagnosis and after treatment is based on the grading system, the PSA level, the tumor-node-metastasis (TNM) classification, and/or the previous treatment history in order to predict potentially lethal PCa and to inform treatment decisions [29]. Some patients with intermediate-risk PCa, and every patient with high-risk PCa, should undergo further imaging studies.

### 4.3. Current Imaging Tools

MRI plays a critical role in the detection of PCa. Multiparametric MRI (mpMRI) has been widely used in the management of localized PCa over the past five years. It can be simply described as a method that is used in order to obtain an ideal three-dimensional (3D) image of the prostate by combining diffusion-weighted (DWI), T2-weighted (T2WI), and dynamic contrast-enhanced (DCEI) images, and, if necessary and available, MR spectroscopy images. mpMRI is an advanced type of MRI scan that provides a more detailed picture of the prostate than a standard MRI scan can. The most clear-cut indications of mpMRI are the patients with increased PSA levels, a history of negative biopsy, and the presence of additional findings supporting its use in active surveillance (discussed in Section 6, “Treatment”) and non-biopsied patients [30]. 

The use of mpMRI can be beneficial in detecting castration-sensitive prostate cancer (CSPC) with a better responsiveness to ADT in patients with negative initial biopsies. However, mpMRI lacks the sufficient resolution to detect PCa with a lower Gleason grade and a smaller volume. Cancerous lesions in the mid and the base gland of the prostate can be detected better, but the detection of apical lesions is not sufficient [31,32]. The interpretation of the mpMRI findings must be carried out according to standardized scoring systems (such as the Prostate Imaging Reporting and Data System (PI-RADS) v2) between different readers [33]. Globally, the application of MRI is recommended before biopsy procedures. A limitation of using the spatial information of suspected lesions that is obtained from MRI for a targeted biopsy is that the MRI-visible lesion is assumed to be the most relevant clinically, which may not be the case.

In the identification of PCa, computed tomography (CT) plays a minor role and is not advised for reasons including poorly defined gland margins and the low resolution of the prostate soft tissue. Although a CT scan is occasionally used for the lymph nodal staging of PCa [34], it poorly performs in the detection of lymph node involvement because of the similar sizes of benign reactive nodes and metastatic nodes [34].

Positron emission tomography (PET) has a significant superiority in the detection of metastatic extraprostatic disease, and there are various tracers for a PET scan for detecting PCa [35]. For instance, 18F-fludeoxyglucose (FDG), 18F-sodium fluoride, 18F-choline, 18F-fluciclovine, 11C-choline, 68Ga-prostate-specific membrane antigen (PSMA), and 117Lu-PSMA are the clinically available tracers [36,37]. FDG performs better in detecting the metastatic lesions than in detecting the primary lesions, which has been attributed to the increased metabolic activity in metastatic lesions. PSMA-PET scans perform better than choline or acetate PET scans, with a higher sensitivity for the diagnosis of positive lymph nodes and distant metastasis. In 2022, the FDA approved 117Lu-PSMA-617 as a radioligand therapy for PSMA-positive mCRPC treatment (NCT03511664) [38]. 

## 5. Genomics

In the past decade, we have gained considerable insights into the genetic basis that underpins distinct PCa subtypes from unparalleled advances in mRNA sequencing, whole-genome DNA sequencing, and proteome profiling [39,40].

Although approximately 90% of PCa cases are found in men without a family history of the disease, PCa appears to run in some families, indicating the existence of genetic factors. Men with first-degree relatives with PCa are known to have a two-fold increased risk of developing PCa [41]. PCa risk is also strongly associated with a family history of any type of cancer. Because almost 9% of men with a diagnosis of PCa have a family history of cancer [9,42,43], the incidence of PCa in these families is believed to be one of the highest among any cancer type.

Over a patient’s lifetime, the tumorigenesis of PCa is believed to have a strong association with the accumulated somatic mutations in the genome of the prostate epithelial cells (Table 1). Nevertheless, unlike advanced metastatic PCa, which has a far higher mutation rate and frequency of copy number alterations (CNAs) [39,40], studies on primary localized PCa did not reveal uniform genomic nucleotide-level signatures [44]. Localized PCa exhibits a relatively smaller number of genomic aberrations than other types of cancer, and the mutation rate is 7- to 15-fold smaller than that reported for melanoma and small-cell lung cancer [45]. Single nucleotide polymorphisms (SNPs) that are associated with PCa incidence have been suggested to be applicable in identifying men for targeted screening (NCT03158922), and in the increased detection of low-risk cancers [46,47,48]. In localized PCa, recurrent SNP driver abnormalities are rare and the only gene, to our knowledge, in which SNPs were reported to predict recurrent events is ATM [44]. Another recent study has revealed that SNPs in AR were not observed in localized disease, that SNPs in TP53 were significantly more prevalent in mCRPC, and that SPOP SNPs were less prevalent in mCRPC [49]. Recent genome-wide association studies (GWAS) and cohort studies have revealed the association of SNP rs11672691 on chromosome 19q13 with the clinical characteristics of aggressive PCa, including the progression of PSA and the development of CRPC [50,51]. The altered binding of HOXA2 to the enhancer elements of PCAT19 and CEACAM21 genes from the rs11672691 GG genotype is associated with a poor prognosis for patients with PCa [50]. The risk variants of rs11672691 and rs887391 result in stronger enhancer activity, which suppresses and activates the long non-coding RNA (lncRNA) isoforms PCAT19-short and PCAT19-long, respectively [51]. However, the functional link between the causation of prostate tumorigenesis and SNPs remains unknown. Our reader can refer to an excellent review article for a summary of the SNPs, with an emphasis on lncRNAs, that are found in PCa [52].

PCa is a C-class tumor that has a limited degree of mutations (3–6% of the primary cancer genome) [9] as most of the PCa-associated genetic changes that are observable in up to a third of localized PCa cases are gene methylation, CNAs, or gene structural rearrangements. Kataegis (which are regions where a large number of highly patterned base pair mutations occur in a small region of DNA), chromothripsis (where thousands of clustered chromosomal rearrangements within confined genomic regions in one or several chromosomes occur in a single event), and chromoplexy (a complex DNA rearrangement that is observed in the genomes of cancer cells) are representative gene structural rearrangements [39,57]. Early PCa typically accumulates CNAs, large-scale genomic structural rearrangements, or both [39,44]. An increase in the genetic instability is known to associate with recurrence and progression, including the metastasis of PCa [45,58,59,60,61].

Major gene alterations include gene fusions of TMPRSS2 with ETS family genes [62], the amplification of MYC oncogene, the deletion and/or mutation of PTEN and TP53 tumor suppressors, and, in advanced PCa, the amplification and/or mutation of AR. Some genes mutate during a person’s lifetime. In fact, the spectrum of mutational burdens dramatically changes in the progression of localized PCa to metastasized PCa [4]. Several gene mutations have been suggested to be responsible for the tumorigenesis of PCa. 

Of the germline mutations in CHK2, PALB2, and RAD51D, the mismatch repair (MMR)-related genes (MSH2, MSH6, and PMS2), and the DNA damage repair (DDR) genes, including ATM, ATR, NBS1, HOXB13, BRCA1, and BRCA2, the mutations in BRCA2 [56] and the HOXB13 [63] genes are the top two mutations that contribute to an eight- and three-fold increased relative risk, respectively [64,65,66]. The most frequently mutated DDR genes in both the germline and the somatic cells of mCRPC are BRCA2, ATM, and CHK2 [56,67]. In addition to the increased lifetime risk of PCa, the germline mutations in BRCA1 or BRCA2 can increase its aggressiveness [68,69,70], with the additional activation of MYC from gene amplification in combination with the inactivation of p53 and PTEN [71,72]. In addition, the mutations in the DDR genes are also increased in the progression of PCa [56]. Ovarian cancer (15%), followed closely by PCa, is the cancer type where the somatic mutations in BRCA are mostly found, with a variety of the frequency of mutations between the population studied, the type and the stage of the cancer samples, and the ethnicity of the patient [73].

The proteins in the homologous recombination (HR) system function in DNA repair, but also in chromatin remodeling, in cell cycle regulation, and in transcriptional activation. In the BRCA mutant cells, gross chromosomal rearrangements are increased [74]. It is important to note that this “genomic scar” is left behind by the loss of HR function, irrespective of which component of the pathway was lost. BRCA2, which is a key RAD51 interactor, is phosphorylated by CDKs and PLK1, recruited to stressed replication forks from a DNA break, and promotes genome stability [75]. It can protect the telomere integrity by the loading of RAD51 during the S/G2 phase [76]. The cells can repair DNA damage before entering mitosis and survive with the phosphorylation of BRCA1, in response to DNA damage, by DNA-damage response kinases, such as ATM, ATR, and CHK1 [77]. BRCA1/2 homozygous deletions are frequent in PCa, in which BRCA2 deletions account for 25% of all BRCA1/2-altered cases [78].

The ETS-related gene (ERG) is generally not expressed in non-malignant primary prostate epithelial cells [79], and one of its roles is to attenuate androgen-regulated transcription. Androgen signaling recruits AR and DNA topoisomerase 2-β (TOP2B) to the breakpoint regions within the ERG and the transmembrane protease serine 2 (TMPRSS2) genes, which are each 3 Mb apart on the same chromosome 21, and subsequent double-strand breaks and gene recombination result in a fusion gene [80,81]. TMPRSS2–ERG is the most common ETS family rearrangement. It can be identified in approximately 50% of PCa cases and accounts for 90% of the total ETS family fusions [82,83]. The involvement of the ERG in gene translocation (EWS-ERG and TLS/FUS-ERG) and the high expression of ERG are implicated in cancer, including Ewing’s sarcoma and acute myeloid leukemia, in addition to PCa [79]. Normal prostate tissue generally does not show TMPRSS–ERG fusions [79,84]. Androgen stimulation in the prostate tissue was found to mediate a high expression of the fusion gene of the AR-responsive TMPRSS2 gene, and the ERG was proposed to increase the oncogenic signaling from its reciprocal suppression of AR, which may ultimately result in a resistance to ADT and the induction of the EZH2-mediated dedifferentiation of PCa cells [85]. Furthermore, multiple studies have demonstrated that TMPRSS–ERG has multiple protumoral functions [86,87,88].

Although several studies have suggested its association with a poorer prognosis [89,90], other studies have also revealed that this gene fusion is actually not related to the prognosis of the patient [82,91,92]. The significance of TMPRSS2–ERG fusion in the tumorigenesis of PCa remains unclear. The rates of TMPRSS2–ERG fusion differ among different race and geographical groups, with a wide range of 7–83% [82,93,94]. Because of the extremely high rate of interfocal and intrafocal ERG heterogeneity in patients with PCa [95], the conventional classification of these patients into “fusion type” or “non-fusion type” may not reflect the actual tumorigenic processes or the patients’ prognosis. Patients of an Asian heritage have an extremely small number of TMPRSS2–ERG fusions. FOXA1 is a transcription factor (TF) that is required for the development and the maintenance of the epithelial cells in the prostate, with a role as a pioneer factor to open the closed chromatin for AR [96]. In Chinese patients, FOXA1 mutations, not ETF fusions, are found to be the most prominent PCa signature [57]. A general consensus is that, without a concomitant loss of function of additional tumor-suppressor genes [97,98], the ERG status itself does not necessarily predict the recurrence or the survival rate, although its status may reflect the pathological stage [82].

We have recently found that the cerebral cavernous malformation1 (CCM1) gene is transcriptionally activated, independently with CNA, in advanced PCa, and that CCM1 upregulates YAP/TAZ and AR signaling [99]. Genomic alterations are found in the PCa target common cancer pathways (Ras/Raf, AR, cell cycle, WNT, Hippo-YAP/TAZ, p53, DNA repair gene, Notch, Myc, TGF-β, and Nrf2), although the component genes are not altered at an equal frequency. Metastatic PCa shows a much higher mutational burden [57]. Due to the heterogeneity within a single focus, and between foci, as well as polyclonal subpopulations in metastatic foci, the dynamics of the resistant clones with therapy indicate that the preexistent clonal populations may be responsible for a common resistance to the therapy and the progression of PCa [17].

Epigenetic changes, including DNA methylation, non-coding ribonucleic acids, and histone modifications, can contribute to the initiation and the progression of PCa. The hypermethylation of promoter DNA is involved in DNA repair, hormonal response, signal transduction, the cell cycle, apoptosis, and cell adhesion [100]. DNA hypomethylation is more frequently observed in the late phase, such as metastasis, rather than in the early stage of PCa. DNA hypomethylation is involved in the increased expression of genes coding urokinase-type CYP1B1, HPSE, and PLAU [101]. ConfirmMDx, which is a tissue-based DNA methylation assay, evaluates the methylation of the following three genes: APC, GSTP1, and RASSF2 [102]. Multiple studies have supported that this assay can be applied in cases with suspected PCa with a negative biopsy [101,103]. DNA methylation has been suggested as a circulating biomarker in mCRPC [104,105,106].

Cistrome refers to the genome-wide location of the regulatory elements that are associated with TF binding sites. During prostatic transformation and disease progression, the cistrome of the key regulatory factors that are involved in PCa etiology are substantially reprogrammed, resulting in a global alteration of AR transcriptional signatures. The AR cistrome is drastically altered in the progression of PCa from normal epithelial cells to localized PCa [107], and further to metastatic PCa [108]. It has been suggested that the metastatic AR cistrome reactivates the decommissioned developmental programs of the prostate. The cistrome of AR is reprogrammed by other TFs, such as ERG, FOXA1, GATA2, HOXB13, and MYC. Chromatin remodeling factors, such as SWI/SNF complexes and CHD1 helicase, also alter the AR cistrome during the disease progression. The upregulation of EZH2, which is a catalytic core subunit of PRC2, is associated with an advanced stage and a poor prognosis of PCa. EZH2 contributes to the expression of the AR transcriptional signatures [109] and co-occupies the reprogrammed AR cistrome to transcriptionally modulate the stem cell and neuronal gene networks [110]. The reprogramming of the AR cistrome is also observed with the acquisition of resistance to second generation antiandrogens abiraterone acetate or enzalutamide [110,111]. The alteration of the AR cistrome with the acquisition of resistance to the second generation antiandrogens indicates another resistance mechanism that the reprogramming can provide cancer cells the opportunity to develop AR-independent mechanisms of tumor growth.

## 6. Treatment

Active surveillance is carried out in order to monitor low-grade, slow-growing localized PCa until the patient’s doctor determines that further treatment is necessary in order to stop the disease within a curable stage, rather than treating it straight away The purpose of active surveillance is to avoid complications and the overtreatment of favorable, low- or intermediate risk PCa with low risk of metastasis and mortality [4]. It is important that clinicians detect any switching to a higher risk cancer that requires treatment from a thorough clinical assessment. Suitability for active surveillance is based on risk stratification with PSA, DRE, life expectancy, cancer staging, and biopsy information. Many centers use MRI scans as an additional test before inclusion in active surveillance. Overlaying MRI characteristics and genomic markers in order to improve risk stratification are now studied [112,113]. 

**Figure 1:**
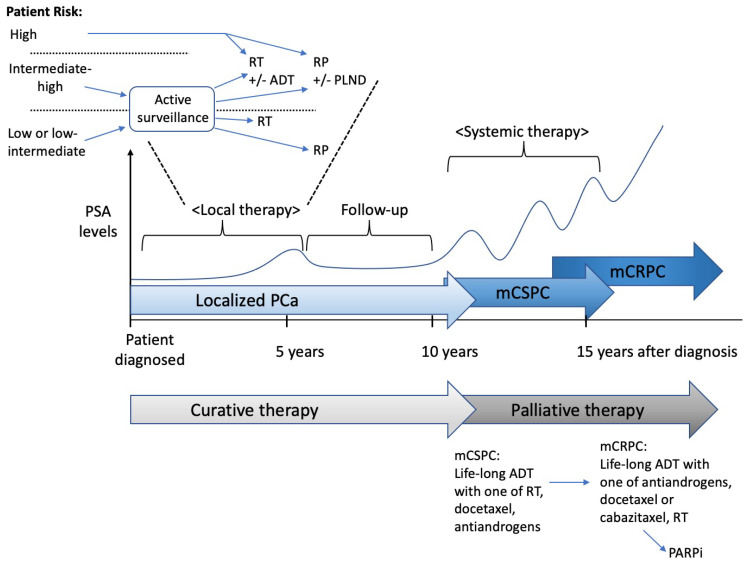
Management overview of prostate cancer. Patients with localized prostate cancer are predominantly managed with either active surveillance or local treatment. The division into low-, intermediate-, and high-risk prostate cancer uses multiple parameters, such as the number of cancer-positive biopsy cores, the length of tumor architecture in the cores, molecular signatures, and imaging results, and inform the decision between active surveillance, single modality therapy, or multimodality therapy. Prostate-specific antigen (PSA) level since diagnosis, indirectly representing the tumor burden, increases in patients whose prostate cancer fails to respond to local and systemic therapies in the progression to metastatic disease. The aggressive prostate cancers are associated with the progression from localized to metastatic castration-sensitive prostate cancer (mCSPC) and metastatic castration-resistant prostate cancer (mCRPC). The treatment approach is switched from curative to palliative care in the disease progression. ADT: androgen deprivation therapy, PARPi: poly(adenosine diphosphate–ribose) polymerase inhibitor, PLND: pelvic lymph node dissection, RP: radical prostatectomy, RT: radiotherapy.

For localized PCa, local treatment with radiation or surgery is potentially curative (Figure 1). One of the laparoscopic radical prostatectomies, robot-assisted radical prostatectomy (RARP) or an open radical prostatectomy (RP), is chosen for the surgical treatment. An increasing tendency for the use of a radical prostatectomy has been reported in the USA, even for patients with high-risk PCa [114]. Despite the growing concerns and the various recent warnings that the actual benefit of RARP use is unclear [115,116], it is frequently used to treat localized PCa [117]. In the USA, RARP is the most common surgical approach for PCa [118], and by 2014, it accounted for up to 90% of the total radical prostatectomies that were conducted [119].

In fact, radiotherapy is reported to be curative in 60% of men with localized PCa [120]. Compared with RP, the potential benefits of initial radiotherapy include its availability for surgically difficult patients or unresectable cancer lesions and the avoidance of substantial complications, such as urinary incontinence and erectile dysfunction, resulting from RP. To summarize, the current surgery and radiation therapies are not ideal when only partial or subtotal tissue removal is required instead of radiation or full-organ removal.

For controlling metastatic disease, the reduction in the circulating androgen levels through chemical castration is the foundation of systemic therapy. As mentioned earlier, the proliferation and the differentiation of the prostate epithelial cells are dependent on the AR activity. In CSPC, following androgen ligand binding to AR, activated AR dimerizes in the nucleus, which binds to the androgen-response elements in the AR-regulated downstream genes and upregulates their expression. Localized PCa almost universally responds to ADT. Luteinizing the hormone-releasing hormone analogs in order to decrease the LH levels before the progression of PCa leads to the termination of testicular testosterone production, which is a medical castration (i.e., ADT). Antiandrogens were originally given with ADT in a combined androgen blockade. Almost all patients with PCa ultimately develop CRPC, which is refractory to ADT, within 12–18 months and have a mean survival of 14–26 months (Figure 1) [121]. The resistance mechanisms that are responsible for abnormal changes in AR signaling are discussed in Section 7, “Mechanisms of resistance to antiandrogen therapy”. 

Second-generation antiandrogens, such as enzalutamide, darolutamide, apalutamide, and abiraterone acetate, and radiotherapy, including external beam radiation therapy (EBRT) with X-ray beams and radiopharmaceuticals, including Ra-223 and 117Lu-PSMA-617 [38], and immunotherapy, including sipuleucel-T, dostarlimab, and pembrolizumab, have been approved and are available for treating patients with mCRPC [122,123]. There are multiple combination therapeutic strategies that have been tested. Enzalutamide plus abiraterone acetate was tested in patients undergoing resistance to enzalutamide (NCT01995513) [124]. Abiraterone acetate with prednisone and apalutamide has also been safely tested successfully in a clinical trial with mCRPC (NCT02257736) [125]. ADT with apalutamide was successfully tested in mCSPC (NCT02489318) [126]. Abiraterone acetate plus prednisone, combined with ADT, significantly lengthened time of progression-free survival (NCT01715285) [127]. Ra-223 with abiraterone acetate and/or prednisone in mCRPC was not successful in improving the skeletal event-free survival (NCT02043678) [128]. In comparison with abiraterone alone, abiraterone acetate in combination with olaparib showed an improved clinical benefit and also more adverse effects [129]. Pembrolizumab was tested with enzalutamide in mCRPC (NCT02312557) [130]. EZH2 inhibitors are under investigation with abiraterone and enzalutamide (NCT03480646), or with the AR antagonist (NCT03741712), in the treatment of mCRPC. 

Approximately 23% of mCRPC tumors harbor loss-of-function germline or somatic mutations in the DDR genes, such as BRCA1, BRCA2, ATM, and CHK2 [40]. Cells with defects in the DNA double-strand break (DSB) repair genes, such as HOXB13, BRCA1, BRCA2, CHK2, and ATM, may have a deficiency in the homologous repair pathway, which leads to high CNAs and increased damage from ionizing radiation, DNA strand intercalators, such as platinum, and poly(adenosine diphosphate–ribose) polymerase inhibitors (PARPi), potentially stratifying a subset of patients who may benefit from these non-standard therapies [53,73,131]. The PAR chains on the target proteins near single-stranded DNA break, which are synthesized by PARP1, recruit other DNA repair effectors in order to complete DNA repair [73]. Olaparib, which is a PARPi, was found to improve the progression-free survival of patients with mCRPC who have at least one alteration in the BRCA1, BRCA2, ATM, or FANC gene from the induction of synthetic lethality in comparison with enzalutamide or abiraterone [132,133,134]. The only PARPi to be investigated in monotherapy in a phase three trial for advanced PCa is Olaparib [73]. SPOP encodes a subunit of a Cullin RING E3 ubiquitin ligase, and its mutation prevents the degradation of the ERG and the AR [135,136,137]. Recurrent missens mutations in SPOP are observed in ~10% of localized PCa [57,138]. PDK1 regulates AKT. Recently, the SPOP mediated degradation of PDK1 and the oncogenic roles of loss-of-function mutations of SPOP in the tumorigenesis of PCa through activating the AKT kinase were reported [139]. Another report showed that SPOP mutations increased the sensitivity to AR inhibition with bicalutamide, compared to controls, indicating an improved response to AR targeted therapies [140]. Because the SPOP mutation affects DSB repair, it is associated with genomic instability and sensitizes the cancer cells to DNA-damaging agents, such as PARP inhibitors [141]. 

Although whether adding PARPi to the current standard form of treatments for localized or locally advanced PCa will improve the treatment efficacy currently remains unclear, PARPi is currently considered for those patients who have pretreated mCRPC and distinct deleterious mutations in the HR gene. Mutations in the BRCA2 gene, especially homozygous deletions, appear to best predict the response to PARPi [142]. Traditionally, BRCA testing has been conducted with germline DNA when a familial aggregation of cancer is suspected. BRCA testing is now recommended for all metastatic PCa patients, regardless of their personal or family history of cancer [143]. However, somatic testing is associated with higher rates of sequencing failure [132]. For this reason, we need a consensus protocol for high-quality affordable biomarker testing.

Other than the mutations in BRCA1, BRCA2, or ATM, the types of DDR mutations that may confer vulnerable sensitivity to PARPi and benefit patients remain to be determined. Our reader can refer to an excellent review article for a summary of the current recommendations for genetic testing based on international clinical guidelines [73]. 

## 7. Mechanisms of Resistance to Antiandrogen Therapy

AR activity is not only essential for PCa development, but it is also the major driver of progression to the castration-resistant stage, with current therapy targeting AR signaling [9]. The abnormal changes in AR signaling during cancer progression to CRPC result from the amplification and/or the overexpression of the AR gene, sustained AR signaling by the binding of ligands other than androgen (promiscuous activity), and point mutations that result in mutant (truncated) or splice variants of AR with constitutive activity. The mechanism of resistance also includes the restoration of AR signaling without AR alterations, including intracrine androgen biosynthesis and AR cofactor alterations in the tumor microenvironment. In the male body, >95% of testosterone is produced in the testes. However, prostatic, adrenal, and intratumoral androgens also have a considerable role in resistance because a small overexpression of AR can compensate for the lack of androgen with withdrawal, sensitizing the cancer cells to small amounts of androgen ligand in order to sustain AR signaling [122,144]. A comparison of CRPC and CSPC showed that a subset of CRPCs can persistently metabolize the adrenal androgens into stronger testosterone. The cancer cells of both CRPC and CSPC express CYP17A1, which is essential for the synthesis of androgen from pregnenolone and progesterone [145]. CYP17A1 can maintain intratumor androgen levels that are sufficient enough to reactivate AR signaling in CRPC and promote the resurgent growth of the cancer lesion. Moreover, gain-of-function changes in the androgen biosynthesis pathway contribute to this process [146]. In particular, abiraterone acetate, which is a CYP17A1 inhibitor, has been implemented as a second-generation antiandrogen therapy for PCa progression with ADT. In advanced PCa, immunotherapy with immune checkpoint inhibitors have not been successful [147,148]. A recent report showed that T cell intrinsic AR activity represses the IFNγ expression from T cell exhaustion and that AR blockade can directly enhance CD8 T cell functions in order to sensitize the tumor bearing host to an immune checkpoint blockade [149]. These findings indicate a novel resistance mechanism to immunotherapy and how AR activity may modify the T cell repertoire in mCRPC patients. It is possible that resistance mechanisms maintaining the AR activity (from maintaining intratumor androgen levels) in the tumor microenvironment may also impair the immune checkpoint blockade.

## 8. Neuroendocrine Prostate Cancer

Neuroendocrine prostate cancer (NEPC), which is a subpopulation of t-CRPC, is a rare and lethal subtype of PCa, occurring in approximately <2% of patients with PCa, with a 10% 5-year survival rate [150]. The prevalence of NEPC is increasing as patients with metastatic PCa receive newer antiandrogen treatments [151]. NEPC is observed in 20–25% of patients with CRPC, with recurrence during ADT [123]. NEPC has features that are common to smell-cell lung cancer [152], and it exhibits the secretion of neuronal factors, the expression of neuronal markers, distinct changes in DNA methylation [153], and a loss of dependence in AR signaling [9]. Although several potential therapeutic approaches have been discussed [9,123], NEPC has no effective targeted therapy that is approved by the FDA. Tremendous efforts, with an emphasis on the morphological variations, have advanced the classification of NE lesions in PCa. In 2013, the Prostate Cancer Foundation proposed NEPC to be classified as follows: (I) a usual prostate adenocarcinoma with NE differentiation, (II) an adenocarcinoma with Paneth cell NE differentiation, (III) a carcinoid tumor, (IV) small-cell carcinoma (SCC), (V) large-cell NE carcinoma (LCNEC), (VI) mixed (small- or large-cell) NE carcinoma—acinar adenocarcinoma [154,155]. Recently, based on the expression of neuronal TFs, ASCL1 and NEUROD1, two distinct NEPC subtypes were identified [156].

Because the inability to correctly diagnose NEPC (i.e., the differentiation of NEPC from CRPC) is a fundamental and serious problem, current patient management remains generally not possible with the genetic profiling of the tumor. This is partially due to a wide range of driver mutations (genetic heterogeneity) that are responsible for tumorigenesis.

NEPC originates clonally from a CRPC precursor, rather than from the selection of neuroendocrine clones [153]. It suggests a divergent evolution of NEPC from one or more CRPC cells. It was demonstrated that AR-independent CRPC that shares NEPC-specific molecular changes represents high-risk PCa for progression or in transition toward NEPC [153]. In contrast, many studies have reported focal neuroendocrine differentiation in 30–100% of prostate adenocarcinomas before the initiation of any treatment [157]. Therefore, the identified molecular mechanisms for acquiring an NE phenotype still remain incomplete. 

TFs play a critical role in prostate cancer cell lineage plasticity. A recent study reported that NEPC could be derived from the prostate adenocarcinoma cells of various pathological stages, and the entire process is orchestrated by selective lineage-specific TFs, such as ASCL1 (common TF), NKX2-2 (NE1-specific, early stage), POU3F2, and SOX2 (NE2-specific, late-stage) [150]. In the same study, a stage-specific high expression of TFs was identified in the transdifferentiation of NE from its adeno precursor. 

It has been shown that the overexpression of AKT1 and N-Myc in human prostate epithelial cells give rise to NEPC [158]. The inhibition of N-Myc with MLN8237 showed progression-free survival of 2.3 months in NEPC (NCT01799278). EZH2, which is an epigenetic modulator, co-operates with lineage-guiding TFs in order to epigenetically control the expression of genes and the specification of lineage. It has been shown that EZH2 complexes directly with N-Myc in order to transcriptionally repress the genes that enforce an AR-driven adenocarcinoma state in NEPC [159], and that EZH2 knockdown leads to the de-enrichment of neuronal-associated pathways in NEPC organoids [160]. EZH2 inhibitors are under investigation with abiraterone and enzalutamide (NCT03480646), or with AR antagonist (NCT03741712), in the treatment of mCRPC. ONECUT2 drives the aggressiveness in NEPC, partially through synergizing with hypoxia in order to suppress androgen signaling and induce neuroendocrine plasticity [161]. In addition to the repression of the genes that enforce the epithelial lineage, ONECUT2 directly activates the neuroendocrine lineage markers, such as PEG10, and displaces the AR-dependent growth and survival mechanisms, suggesting its possibility as a potential drug target in mCRPC [162]. 

## 9. Future Studies

PCa is a heterogeneous disease that shows a wide variability in biology and clinical progression. Estimating the degree of risk based on clinical features and distinguishing low-risk localized PCa from aggressive PCa are the central clinical challenges that must be overcome in order to further improve outcomes while adapting the treatment to individual risk profiles and the risk of PCa-specific morbidity and mortality. The global men’s health charity “Movember” commissioned a formal landscape analysis in order to evaluate the current PCa research field and reported 17 research needs [163]. The following top three research needs in the field were agreed upon and prioritized by Movember: the establishment of more specific and sensitive tests to improve the current screening and diagnosis of PCa, the development of indicators to stratify patients with low-risk PCa for correct active surveillance enrollment, and the integration of companion diagnostics into randomized clinical trials for the prediction of treatment responses. The other research needs that were prioritized by Movember were the accurate determination of oligometastatic PCa (PCa with three to five metastatic lesions) with more sensitive and specific molecular imaging in order to reclassify nonmetastatic disease into metastatic disease and the best treatment strategy and the demonstration of the clinical utility of validated liquid biopsies.

The current standard classification into low-, intermediate-, and high-risk PCa increasingly incorporates factors such as the number of positive biopsy cores, the length of the tumor lesions in the biopsy cores, positive imaging results, and various mutational signatures [164]. Clinical and pathological variables, in combination with genomic biomarkers, are useful methods to reduce the practice of unnecessary biopsies, to stratify patients with low-risk PCa from those with high-risk PCa, and to provide and guide personalized treatment options for each patient [165]. Despite the progress with suitable PCa biomarker candidates, only a few have been applicable in a clinical setting. Therefore, we need large-scale multi-institutional studies to validate the cost utility and the efficacy of these new technologies.

Currently, it is not clear as to whether any further improvements to molecular subtyping, such as mutational signatures, will advance risk-adapted management or whether identifying individual molecular subtypes is of prognostic or predictive benefit. However, the current disease management algorithms require further reassessment, and all of the evidence emphasizes the crucial importance of other new therapies that can target pathways other than AR signaling in PCa cells.

We have seen a revolutionary advance in both PCa patient care and the research field in recent years, with a shift from surgical and medical therapy to active surveillance in order to reduce the burden of treatment on health-care services and to improve the patient tolls of PCa that, in some instances, do not require therapy [166]. A limitation of these changes is that some patients will face difficult decisions regarding their treatment options in their life-long treatment workup. Currently, preventative interventions for primary PCa have not been established.

## 10. Conclusions

Major improvements in the guidelines of PSA screening and testing, and the indication of imaging tools, have increased their use in PCa diagnostics. With the advent of our understanding of PCa biology from bedside, genomics, and molecular imaging techniques, it is imminent that our current theragnostic schemes, including the diagnostic modalities, the estimation of cancer progression from indolent to malignant status and therapeutic responses, and the drugs that are used to target non-AR signaling, including DNA repair defects, should be revised and improved.

## Figures and Tables

**Table 1:** Frequency of somatic and germline mutations by prostate cancer stage. Reprinted from the Lancet, 398, Sandhu et al., Prostate cancer, 1075–90 [4], Copyright 2021, with permission from Elsevier. * Castration sensitivity was not defined in this study.

Somatic mutations	Localized (n = 333) [39]	Metastatic, Castration-Sensitive (n = 140) [53]	Metastatic, Castration-Resistant (n = 444) [54] and (n = 101) [55]
*TMPRSS2–ERG* fusion	46.0%	Not reported	41.0% and 43.0%
Other ETS family gene fusions	14.0%	Not reported	10.0% and 15.0%
SPOP mutation	11.0%	11.0%	5.0% and 6.0%
CHD1 deletion	7.0%	6.0%	23.0% and 33.0%
*FOXA1* mutation	4.0%	10.0%	9.0% and 19.0%
*PTEN* deletion (homozygous)	17.0%	17.0%	32.0% and 45.0%
*TP53* mutation or deletion	8.0%	30.0%	40.0% and 57.0%
*RB1* deletion (homozygous) or mutation	1.0%	7.0%	12.0% and 13.0%
*PI3K* mutation	3.0%	5.0%	5.0% and 5.0%
*AKT* mutation	1.0%	2.0%	1.0% and 2.0%
*BRCA1* mutation or deletion	1.0%	1.0%	1.0% and 2.0%
*BRCA2* mutation or deletion	3.0%	7.0%	10.0% and 11.0%
*ATM* mutation	1.0%	2.0%	1.0% and 2.0%
*CDK12* mutation	2.0%	6.0%	3.0% and 7.0%
Mismatch repair mutation or microsatellite instability	5.0%	5.0%	4.0% and 5.0%
*APC* deletion	5.0%	13.0%	8.0% and 9.0%
*CTNNB1* mutation	2.0%	6.0%	4.0% and 6.0%
*MYC* gain-of-function	7.0%	6.0%	23.0% and 33.0%
*AR* amplification or mutation	1.0%	4.0%	59.0% and 70.0%
Germline mutations	Localized (n = 499) [56]	Metastatic * (n = 692) [56]	
*BRCA1*	0.6%	0.9%	..
*BRCA2*	0.2%	5.3%	..
*ATM*	1.0%	1.6%	..
*CHEK2*	0.4%	1.9%	..
*PALB2*	0.4%	0.4%	..
*RAD51D*	0.4%	0.4%	..
Mismatch repair (Lynch syndrome)	0.6%	0.6%	..

## Data Availability

Not applicable.
